# Management of Recurrent Biliary Anastomotic Stricture After Pancreaticoduodenectomy Using a Bioabsorbable Stent: A Case Report

**DOI:** 10.7759/cureus.108418

**Published:** 2026-05-07

**Authors:** Daniel Ernesto Mejia Isaza, Luis Fernando Rivera Velásquez, Diana Carolina Lopez Gonzalez, Carlos Andres Zapata Quintero

**Affiliations:** 1 Vascular Surgery, Universidad de Antioquia, Medellín, COL; 2 Interventional Radiology, Clínica Angiosur, Medellín, COL; 3 Interventional Radiology, Clínica Especializada EMMSA, Medellín, COL; 4 Pediatrics, Universidad Cooperativa de Colombia, Antioquia Campus, Medellín, COL; 5 Radiology, Universidad de Antioquia, Medellín, COL

**Keywords:** biliary anastomosis, biliary stricture, bioabsorbable stent, interventional radiology, pancreaticoduodenectomy

## Abstract

Bilioenteric anastomotic strictures are a late complication of pancreaticoduodenectomy, often leading to recurrent biliary obstruction and cholangitis. Due to altered postoperative anatomy, percutaneous transhepatic interventions frequently represent the preferred therapeutic approach when endoscopic access is limited.

Standard treatment consists of balloon dilation and placement of plastic or metallic stents, although these are associated with restenosis, occlusion, and the need for repeat procedures or device removal. Bioabsorbable stents have emerged as a potential alternative, providing temporary luminal support while eliminating the need for retrieval.

We report a 70-year-old male with a history of pancreaticoduodenectomy and recurrent bilioenteric anastomotic stricture, previously managed with an internal-external biliary drain. A percutaneous transhepatic approach was performed under general anesthesia, including recanalization with a hydrophilic guidewire, balloon dilation, and deployment of a bioabsorbable stent. Final cholangiography demonstrated adequate biliary drainage without residual stenosis.

Bioabsorbable stents may offer an effective, minimally invasive option in selected patients with complex biliary anatomy, potentially reducing the need for repeat interventions. Further studies are required to assess long-term patency and clinical outcomes.

## Introduction

Biliary strictures represent a well-recognized late complication following pancreaticoduodenectomy, with significant implications for patient morbidity and healthcare utilization [1-4]. These strictures, particularly those occurring at the bilioenteric anastomosis, can lead to recurrent cholestasis, episodes of cholangitis, and progressive deterioration of liver function if not treated in a timely manner [1-3]. The management of these conditions has evolved considerably in recent decades, shifting from predominantly surgical approaches to minimally invasive techniques, which are currently considered first-line therapy in most clinical scenarios [2,5].

Minimally invasive strategies include both endoscopic and percutaneous approaches, with the choice largely determined by postoperative anatomy. In patients who have undergone pancreaticoduodenectomy, altered gastrointestinal anatomy frequently limits endoscopic access to the biliary tree, thereby increasing the relevance of percutaneous procedures [2,5]. Among these, balloon dilation combined with stent placement constitutes the cornerstone of treatment. However, conventional stents, whether plastic or metallic, present limitations such as the need for multiple interventions, risk of occlusion, and, in some cases, challenges related to their removal [5].

In this context, bioabsorbable stents have emerged as an innovative therapeutic alternative. These devices are designed to provide temporary structural support to the biliary duct, maintaining luminal patency during the critical period of tissue remodeling and gradually degrading over time, thereby eliminating the need for removal [6,7]. This characteristic offers a potential advantage by reducing the number of required procedures and improving patient comfort.

## Case presentation

A 70-year-old male with a history of pancreaticoduodenectomy (Whipple procedure) presented with recurrent bilioenteric anastomotic stricture. During the index surgery, gastrointestinal reconstruction was performed using a pancreaticojejunostomy and a hepaticojejunostomy. The pancreaticojejunostomy was created in an end-to-side fashion between the pancreatic remnant and a defunctionalized Roux-en-Y jejunal limb using a duct-to-mucosa technique. The hepaticojejunostomy was constructed by anastomosing the common hepatic duct to the same jejunal limb approximately 10-15 cm distal to the pancreatic anastomosis, also in an end-to-side fashion. The patient presented with recurrent episodes of biliary obstruction, evidenced by elevation of liver function tests, including bilirubin, alkaline phosphatase, and gamma-glutamyl transferase, and was temporarily managed with an external biliary drainage catheter.

**Figure 1:**
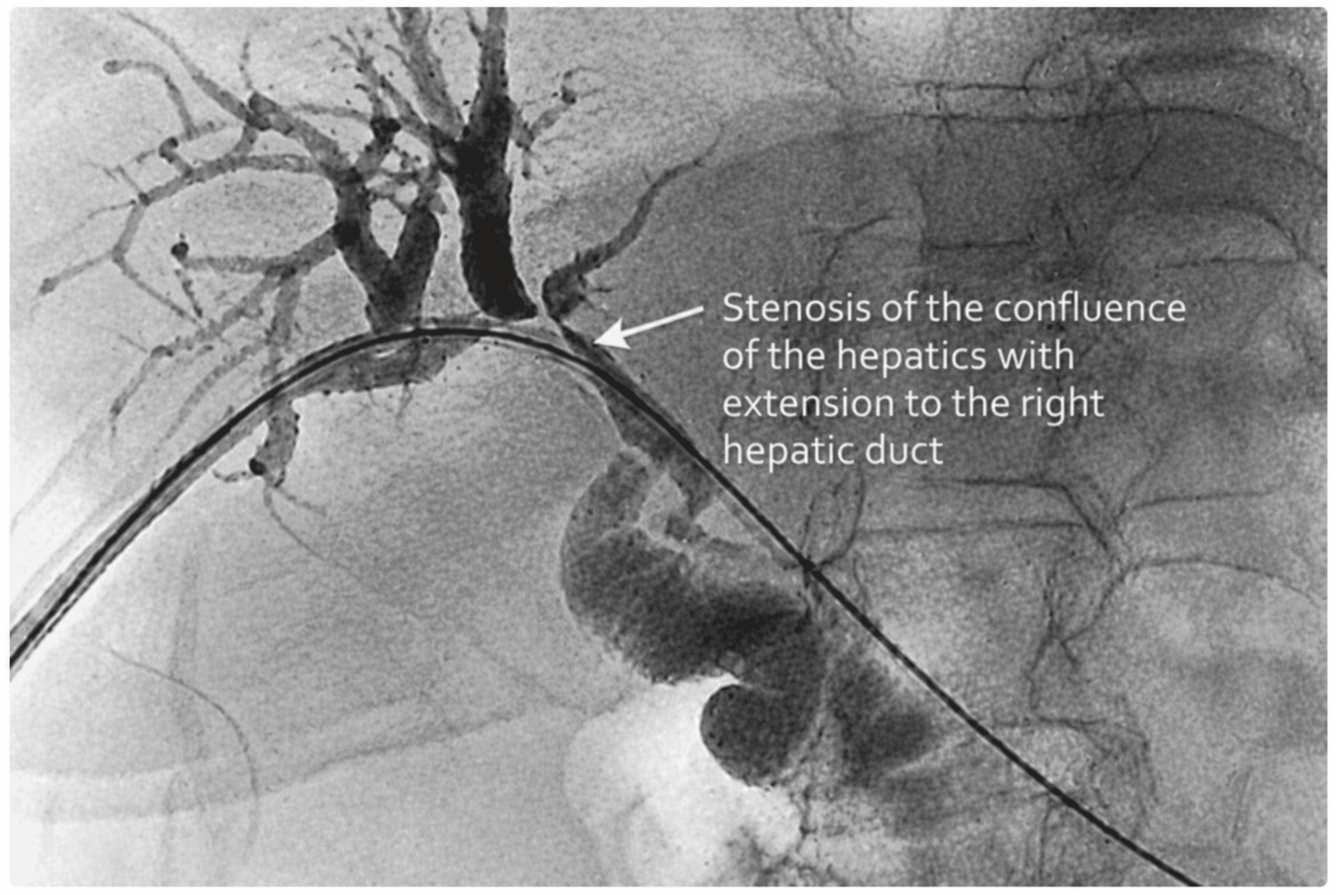
Focal narrowing at the level of the confluence of the right and left hepatic ducts Cholangiographic image demonstrating a focal narrowing at the level of the confluence of the right and left hepatic ducts, suggestive of an intrahepatic biliary stricture. The stenotic segment is identified proximal to the bilioenteric anastomosis, at the point where the contrast column becomes filiform, with associated upstream biliary dilatation. The bilioenteric anastomosis is located distally in the common hepatic duct.

Under general anesthesia, a percutaneous transhepatic approach was performed. The patient already had a pre-existing external biliary catheter through which cannulation was achieved. The existing biliary catheter was then removed, and cholangiography was obtained, demonstrating a focal narrowing at the level of the confluence of the right and left hepatic ducts with extension into the right hepatic duct, with associated upstream biliary dilation (Figure 1).

Recanalization was achieved using a hydrophilic guidewire, which was carefully advanced across the stenotic segment under fluoroscopic guidance. Following successful crossing of the stricture, sequential balloon dilation was performed using 8 × 80 mm and 10 × 80 mm balloons. The procedure was well tolerated, without evidence of contrast extravasation or ductal injury.

**Figure 2:**
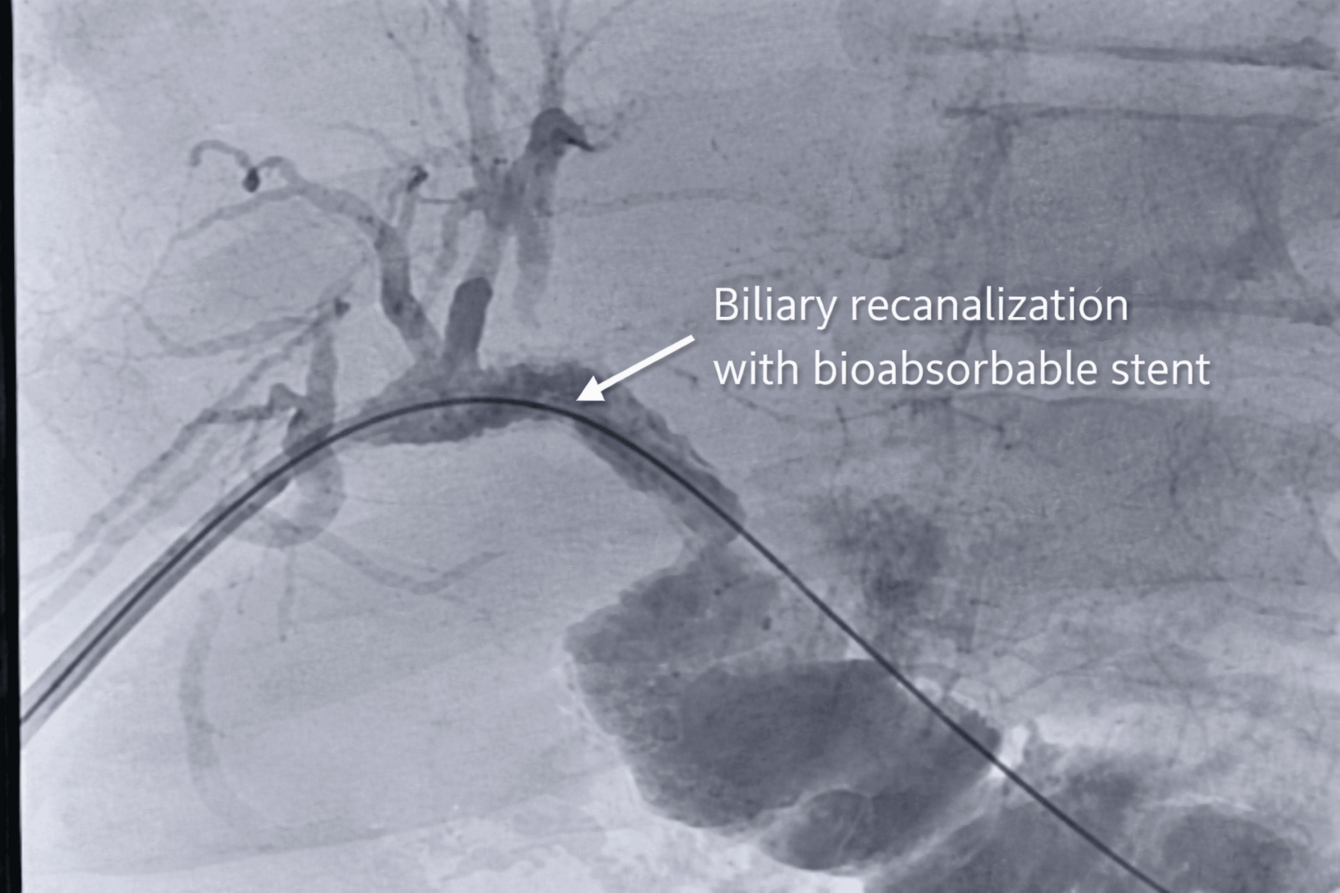
Biliary recanalization after bioabsorbable stent placement Cholangiographic image demonstrating restoration of biliary flow following placement of a bioabsorbable stent. The previously stenotic segment, located at the confluence of the right and left hepatic ducts, shows improved luminal patency, with adequate passage of contrast into the bilioenteric anastomosis and no evidence of residual obstruction.

After adequate dilation, a 10 × 57 mm bioabsorbable stent (Unity-B stent, Q3 Medical, Dublin, Ireland; manufactured in Germany) was deployed across the stenotic segment, ensuring full coverage of the affected area. The stent expanded appropriately and conformed to the ductal anatomy. Final cholangiography demonstrated a marked improvement of the stenotic segment, with restoration of luminal caliber and no significant residual narrowing, confirming effective ductal patency (Figure 2).

The patient’s post-procedural course was uneventful, with no immediate complications. Clinical follow-up showed favorable evolution, with sustained biliary drainage and no recurrence of obstructive symptoms. At one week, liver function tests demonstrated improvement, with total bilirubin decreasing from 5.2 mg/dL to 1.4 mg/dL, direct bilirubin from 3.8 mg/dL to 0.9 mg/dL, alkaline phosphatase from 420 U/L to 210 U/L, and gamma-glutamyl transferase from 380 U/L to 160 U/L. At one month, a biliary ultrasound confirmed appropriate stent caliber and positioning, with no evidence of intrahepatic or extrahepatic biliary stenosis.

## Discussion

Biliary strictures following pancreaticoduodenectomy are a well-documented complication, with a reported incidence ranging from 2.6% to 16.3%, reflecting variability in surgical techniques, patient populations, and duration of follow-up [1-6]. These strictures most commonly develop within the first postoperative year, although delayed presentations several years after surgery have also been reported [1-3,6].

Several risk factors have been associated with the development of biliary strictures in this setting, including a reduced diameter of the common hepatic duct (<4 mm), postoperative bile leak, preoperative biliary drainage, and the use of adjuvant radiotherapy [1-5,7-9]. The underlying pathophysiology is multifactorial and involves ischemia, inflammation, and progressive fibrotic remodeling at the anastomotic site.

Management strategies have evolved significantly, with a shift toward minimally invasive approaches as first-line therapy. Balloon dilation combined with stent placement has demonstrated favorable technical and clinical success rates, ranging from 75% to 92% [1,2,9]. Surgical revision is currently reserved for refractory cases or failure of less invasive treatments, given its higher associated morbidity [2,10].

In patients with altered postoperative anatomy, such as those who have undergone pancreaticoduodenectomy, percutaneous approaches play a central role due to the technical limitations of endoscopic access [2,10]. These techniques allow for both diagnostic evaluation and therapeutic intervention in a single session, contributing to their widespread adoption.

Bioabsorbable stents have emerged as a promising alternative to conventional plastic and metallic stents. These devices are typically composed of polydioxanone (PDO) and are designed to provide temporary radial support while undergoing gradual degradation through hydrolysis over an average period of 9 to 13 weeks, eliminating the need for removal [11,12]. Their application in the biliary system has gained attention since the first reported case of biliary stenting in 2017 [11,12].

Available evidence suggests technical success rates close to 100% and clinical success rates around 80%, with long-term patency rates near 78.9% [13-15]. Compared with multiple plastic stents, bioabsorbable stents offer the advantage of reducing the number of required procedures, which is particularly beneficial in patients with complex anatomy [15].

However, these devices are associated with specific complications. Recurrence of the stricture has been reported in approximately 20% of cases, while stent migration occurs in 3% to 9.4% [14,16,17]. Cholangitis is the most frequently reported complication, although it is typically mild and self-limited [15,18].

From a technical perspective, optimal outcomes depend on appropriate patient selection, adequate pre-dilation, correct stent sizing, and precise positioning to ensure complete coverage of the stenotic segment. Differences in radial force compared with metallic stents may also influence performance in certain clinical scenarios.

In the present case, the use of a bioabsorbable stent allowed effective restoration of anastomotic patency without leaving a permanent implant, potentially reducing the need for future interventions. This approach is particularly advantageous in benign conditions and in patients with complex surgical anatomy.

The Unity-B stent has limited published evidence, primarily restricted to small case series with favorable preliminary outcomes [18]. These findings should be interpreted with caution due to small sample sizes and the lack of comparative studies.

Despite these limitations, current evidence supports the use of bioabsorbable stents as a viable therapeutic option in benign biliary strictures. Further prospective, controlled, and comparative studies are needed to better define their long-term efficacy, safety, and cost-effectiveness relative to conventional treatment strategies [11,14,17].

## Conclusions

Bioabsorbable stents represent a promising therapeutic option for the management of benign biliary strictures following pancreaticoduodenectomy, particularly in patients with complex postoperative anatomy. Their ability to provide temporary ductal support without requiring removal offers a significant advantage over conventional stents and may reduce the need for repeated interventions. However, current evidence remains limited, and further prospective studies are necessary to establish their long-term efficacy, safety, and cost-effectiveness. Careful patient selection and appropriate technical execution are essential to optimize outcomes and minimize complications. In addition, standardized protocols regarding stent sizing, deployment techniques, and follow-up strategies are needed to ensure reproducibility and consistency across different centers.

From a clinical perspective, the use of bioabsorbable stents may represent a valuable addition to the therapeutic armamentarium of interventional radiology, particularly in cases where conventional endoscopic or surgical options are limited or associated with higher morbidity. Future research should focus on comparative studies with plastic and metallic stents, as well as the identification of predictors of treatment success, to better define their role in the management algorithm of benign biliary strictures.
